# Fetal Stem Cells and Skeletal Muscle Regeneration: A Therapeutic Approach

**DOI:** 10.3389/fnagi.2014.00222

**Published:** 2014-08-27

**Authors:** Michela Pozzobon, Chiara Franzin, Martina Piccoli, Paolo De Coppi

**Affiliations:** ^1^Fondazione Istituto di Ricerca Pediatrica Città della Speranza, Padova, Italy; ^2^UCL Institute of Child Health and Great Ormond Street Hospital, London, UK

**Keywords:** fetal cells, muscle dystrophies, cell therapy, placenta, cord blood, amniotic fluid

## Abstract

More than 40% of the body mass is represented by muscle tissue, which possesses the innate ability to regenerate after damage through the activation of muscle-specific stem cells, namely satellite cells. Muscle diseases, in particular chronic degenerative states of skeletal muscle such as dystrophies, lead to a perturbation of the regenerative process, which causes the premature exhaustion of satellite cell reservoir due to continuous cycles of degeneration/regeneration. Nowadays, the research is focused on different therapeutic approaches, ranging from gene and cell to pharmacological therapy, but still there is no definitive cure in particular for genetic muscle disease. Keeping this in mind, in this article, we will give special consideration to muscle diseases and the use of fetal derived stem cells as a new approach for therapy. Cells of fetal origin, from cord blood to placenta and amniotic fluid, can be easily obtained without ethical concern, expanded and differentiated in culture, and possess immune-modulatory properties. The *in vivo* approach in animal models can be helpful to study the mechanism underneath the operating principle of the stem cell reservoir, namely the niche, which holds great potential to understand the onset of muscle pathologies.

## Introduction

The muscular dystrophies are heterogeneous genetic diseases that strongly impair skeletal muscle structure and function. These disorders are caused by mutations in genes encoding for structural proteins essential for muscle integrity, and depending on the alteration, it is possible to distinguish dystrophies due to (i) mutations on a specific gene, such as Duchenne muscular dystrophy (DMD) or on distinct genes, e.g., limb-girdle muscular dystrophies (LGMD); (ii) epigenetic alterations, for instance facioscapulohumeral muscular dystrophy; and (iii) repeat expansion, such as myotonic dystrophy. The primary genetic defect results in a wide range of pathological phenotypes, different for fashion of inheritance, age of onset, severity and distribution of muscle weakness, and speed of progression (Leung and Wagner, 2013).

In particular, DMD is the most prevalent and severe dystrophy that affects skeletal muscle. It is an X-linked recessive disease due to mutations on dystrophin gene, which determines the production of a truncated and non-functional protein. Dystrophin functions as a muscle fiber stabilizer by linking the cytoplasmic actin filaments with the extracellular matrix. The DMD muscle is characterized by continuous fiber damage, which results in an altered regenerative turnover thus leading to the premature exhaustion of muscle stem cell compartment, the development of chronic inflammation, and the infiltration of adipose and fibrotic tissues. In DMD patients, the muscle loss begins very early in childhood starting from lower extremities and leading to progressive difficulty with walking, and finally, up to the confinement in a wheelchair. In addition to loss of mobility, the major disabling co-morbidities are represented by heart disease and respiratory failure that are the most common causes of death (Wilschut et al., 2012; Kharraz et al., 2014). In 1984, Bulfield and co-workers described an X chromosome-linked mutant mouse, also called *mdx*, which mimics the human DMD showing elevated plasma levels of muscle creatine kinase and pyruvate kinase and histological lesions characteristic of muscular dystrophies, although with a milder phenotype (Bulfield et al., 1984). In 2010, Sacco et al. (2010) created a new mouse model where *mdx* mutation was associated with the loss of telomerase (mTR) activity. Indeed, these double-knockout mice display a more severe as well as rapidly progressing phenotype and a greatly reduced life span. In order to allow human cell transplantation for preclinical studies, immunocompromised *mdx* mouse strains have been created, such as *mdx* nude mice (Partridge et al., 1989) and NOD/*Rag1^null^mdx5^cv^* mice (Lapan et al., 2012).

Limb-girdle muscular dystrophies are a family of diseases affecting mainly proximal muscle, in particular shoulders, upper arms, pelvic area, and thighs. These disorders are caused by mutation in various genes encoding muscle structural proteins and present different inheritance patterns; therefore, they consistently differ in terms of age of onset, progress, and severity of disease (Mitsuhashi and Kang, 2012). There are many mouse models for LGMD, according to the variety of involved genes and genetic defects that result in this family of muscle dystrophies. For example, knockout mice for α-sarcoglycan gene accurately resemble the LGMD-2D phenotype (Duclos et al., 1998), a deletion on dysferlin gene produces a LGMD-2B phenotype in SJL-Dysf mice (Bittner et al., 1999), while mice carrying mutations on myotilin gene develop the autosomal dominant LGMD-1A (Liu et al., 2014).

Beside these muscle-specific diseases, there are pathologies that arise from other tissues and consequently affect skeletal muscle. Since the close interplay between nervous system and skeletal muscle compartment, many of these disorders are related to neural degeneration, which causes muscle loss. One example is spinal muscular atrophy (SMA), an autosomal recessive disorder characterized by degeneration of anterior horn neurons and consequent muscle weakness and atrophy. The incidence of SMA is about one in 10,000 live births. It was first described at the end of nineteenth century and only 100 years later the genetic defect was found and localized in the chromosome 5 cared by survival motor neuron (*SMN*) gene (Gilliam et al., 1990; Lefebvre et al., 1995). In humans, there are normally two copies of *SMN* gene, *SMN1* is essential to the pathogenesis, while the number of copies of *SMN2* determines the severity of the disease (Lorson et al., 1998; Feldkotter et al., 2002). Different strategies have been deployed to create murine model of SMA, and using the cre-loxP system the loss of function of *Smn* gene has been directed toward specific tissues, in particular to skeletal muscle. The mouse model *HSA-Cre*, *Smn^F7/F7^* allows to evaluate the effect of *Smn* knockout on muscle since the Cre-recombinase is placed under the control of alpha skeletal actin (Nicole et al., 2003). For this reason, mutant mice display pathological features that mimic a muscle dystrophy phenotype, such as muscle weakness and atrophy and chest deformation with consequent respiratory failure (Cifuentes-Diaz et al., 2001; Salah-Mohellibi et al., 2006).

The research is currently focused on three main therapeutic approaches for muscle diseases. Gene therapy aims to restore the dystrophin protein complex through different strategies, ranging from the development of new vectors capable of delivering efficiently the missing gene to the postmitotic nuclei of the muscle fibers, to exon skipping and the enhancement of the synthesis of proteins such as utrophin. The pharmacological approach is mainly focused on the attenuation of the inflammation (corticosteroids administration) and the treatment of co-morbidities (cardiomyopathy, osteoporosis, respiratory failure). Nowadays, new compounds modulating muscle-specific neuronal nitric oxide synthase (nNOSμ) pathway and TGFβ pathway are under investigation (Berardi et al., 2014). The cell therapy approach is devoted to functionally rescue the tissue through cell delivery; so far, in place of muscle satellite cells, other cells isolated from embryonic (Filareto et al., 2013) and adult [pericytes, bone marrow, mesangioblasts (Ferrari et al., 1998; Gussoni et al., 1999; Sampaolesi et al., 2006; Dellavalle et al., 2011)] sources or induced pluripotent stem (iPS) cells (Darabi et al., 2012; Tedesco et al., 2012) have been shown to contribute to muscle regeneration. However, except for embryonic cells, a real integration of administered cells in muscle niche has barely been observed. Recently, mesenchymal stem cells (MSCs) from human fetal blood (Chang et al., 2006) and skeletal muscle progenitors from mouse embryo (Sakai et al., 2013) have been used in muscle regeneration. Several studies demonstrated that fetal tissues are abundant sources of MSCs, including term placenta, fetal bone marrow, blood, lung, liver, and spleen (in ‘t Anker et al., 2003; Fukuchi et al., 2004), but the most available and easy sources to obtain fetal stem cells are placenta, cord blood, Wharton’s jelly, and amniotic fluid (AF).

## Fetal Stem Cells and Muscle Regeneration

Research on fetal stem cells and muscle is quite recent. Placenta, cord blood, and AF represent an easy source of stem cells without ethical concerns and are immune-privileged tissues (Toda et al., 2007; Wang et al., 2009; Di Trapani et al., 2013). Placenta has been primarily used as a membrane to treat burns, injuries, and skin ulcers (Bujang-Safawi et al., 2010) and it has been studied also to treat lung fibrosis (Cargnoni et al., 2009) and inflammatory corneal diseases (Dekaris and Gabric, 2009). Cord blood has been considered mainly as a source of hematopoietic cells (Cairo and Wagner, 1997), although new field of applications such as diabetes are open (Zhao et al., 2012). Amniotic fluid stem (AFS) cells recently found applications from the heart (Bollini et al., 2011) to the kidney (Rota et al., 2011) and the lung (Carraro et al., 2008).

This review will evaluate the use of cells from the above mentioned sources in muscle regeneration (Table 1), focusing on both the limits and future potential for therapy.

**Table 1:** **Different fetal stem cells displayed myogenic differentiation ability**.

Perinatal sources	Mouse model/injury	Engraftment	Reference
**HUMAN ORIGIN**			
UCB (CD34^+^)	Sjl-Disf	Yes, 12 weeks post injection	Kong et al. (2004)
UCB (MSCs, CD133^+^)	Balb/cA-nu: hindlimb ischemia damage	No	Koponen et al. (2007)
UCB (MSCs)	Mdx	Yes, Dys^+^ fibers 45 days post local injection	Nunes et al. (2007)
UCB (CD34^+^)	CD1: hindlimb ischemia damage	MyoD^+^ cells	Pesce et al. (2003)
Wharton’s jelly	Lewis rat: bupivacaine injury	Yes, 15 days post local injection	Conconi et al. (2006)
Placenta	*In vitro* only	Lentiviral transfection with MyoD	Akizawa et al. (2013)
Placenta	SCID/mdx	Yes, 4 weeks post injection	Kawamichi et al. (2010)
Placenta (CD146^+^, CD34^+^, CD146^−^CD34^−^)	SCID/mdx	Yes, 4 weeks post injection	Park et al. (2010)
AF c-Kit cells	SCID	No	Gekas et al. (2010)
AFS cells	BALB/cSlc-nu	Yes, 21 days post injection	Kim et al. (2013)
AFS cells	NOD/SCID	Yes, 4 weeks post injection	Ma et al. (2012)
**MOUSE ORIGIN**			
AFS cells	HSA-Cre, Smn^F7/F7^	Yes, 1 and 15 month post injection and after secondary transplant	Piccoli et al. (2012)

### Cord blood and Wharton’s jelly cells

Umbilical cord connects the fetus to the placenta; it develops from the body stalk of the embryo and contains blood vessels and Wharton’s jelly, surrounded by the amnion. Umbilical cord blood (UCB) supplies the developing fetus of nutrients and oxygen through the umbilical vein.

Umbilical cord blood possesses three main types of stem cells that are of interest in regenerative medicine: (1) hematopoietic stem cells, (2) MSCs, and (3) non-hematopoietic multipotent stem cells characterized by the expression of SSEA-4, the transcription factors OCT4, SOX2, and NANOG usually expressed by pluripotent stem cells (McGuckin et al., 2005, 2008; Kucia et al., 2007; Zuba-Surma et al., 2009). The blood vessels of the cord blood are insulated with the gelatinous connective tissue called Wharton’s jelly that prevents vein compression (Ferguson and Dodson, 2009). The cells isolated from this biological source share properties with MSCs from UCB such as differentiation ability (Anzalone et al., 2010). Upon *in vitro* muscle differentiation, CD105^+^ cells form Wharton’s jelly have been injected in injured rat *Tibialis anterior* muscle and after 15 days a mild muscle regeneration has been detected (Conconi et al., 2006).

The involvement of UCB in therapeutic application was reported since 1972, when clinicians treated one case of lymphoblastic leukemia (Ende and Ende, 1972). Nowadays, the translational potential of the clinical applications of UCB stem cells is increased due to important advantages such as the ease to recover the cells right after birth without any risk for the donor, the lack of ethical issues, the low onset of graft-versus-host disease (GVHD) (Broxmeyer et al., 1989; Ballen, 2005; Schoemans et al., 2006; Brunstein et al., 2007; Hwang et al., 2007; Broxmeyer, 2010), and finally the high proliferation rate and long telomere maintenance (Kim et al., 1999; Pipes et al., 2006). Starting from 2003, Pesce and co-workers demonstrated muscle amelioration after injection of CD34^+^ UCB cells in injured adductor muscle and new MYOD^+^ cells of UCB origin (Pesce et al., 2003) in a CD1 mouse model of hindlimb ischemia. Later, in 2007, Koponen and colleagues, using ischemia damage in the immunocompromised BALB/cA-nu mouse, analyzed both endothelium and muscle after local injection of several cell types, namely UCB CD133^+^, CD34^+^, MSCs, VEGF-D, or eGFP transduced cells, highlighting that the skeletal muscle regeneration was an indirect effect of cytokines, such as VEGF and FGF, released by the injected progenitor cells (Koponen et al., 2007).

In the *mdx* mouse, Nunes and colleagues were able to find a small number of human dystrophin-positive fibers 45 days after injection of CD34^+^ UCB cells in quadriceps muscle. In the murine model of LGMD-2B, the SJL-Dysf mouse, whole UCB, and CD34^±^ subgroups are engrafted in muscles after systemic injection (Nunes et al., 2007), and expression of dysferlin and human dystrophin was detected 12 weeks post injection of different subpopulation of UCB cells, although in a small amount (Kong et al., 2004).

### Placenta stem cells

The placenta develops at the feto-maternal interface and is a temporary organ that grows during pregnancy. It is formed by fetal (amnion and chorionic plate) and maternal (decidua) portions and contains different types of cells. Indeed, the placenta is rich in stem/progenitor cells. While in the amnion human amniotic epithelial cells, which express embryonic stem cell markers, have been isolated (epiblast-derived pluripotent/multipotent stem cells) (Prusa and Hengstschlager, 2002; Miki et al., 2005, 2007), the placenta MSCs have been separated not only from amnion, but also chorion and decidua (Huang et al., 2009; Pozzobon et al., 2014). These cells share with the adult MSCs the spindle-shape appearance and the property to adhere to plastic and to expand. Moreover, placenta MSCs expand faster *in vitro* and are more immunosuppressive and less immunogenic than the adult MSCs (Battula et al., 2008; Brooke et al., 2008). Recently, a specific subgroup of cells isolated from the human placenta villi and relevant for muscle regeneration, namely the perivascular multipotent mesenchymal progenitor cells, have been characterized by Park et al. (2010). In this research, they studied the angiogenic and myogenic potential of the CD146^+^ CD45^−^ CD34^−^ CD56^−^ cells (pericytes fraction) and CD146^−^ CD45^−^ CD34^−^ CD56^−^ cells (non-pericytes fraction). When these cell populations were intramuscularly implanted into damaged *Gastrocnemius* of immunodeficient dystrophic mice, fibers positive for human dystrophin were found at the periphery of the damaged area. The combination of perivascular progenitors together with the multipotent MSCs isolated from the placenta enhanced the migration and regeneration capacity of the placenta stem cells demonstrating that this extra-embryonic tissue is a reservoir of stem/progenitor cells with myogenic potential. The *in vitro* myogenic differentiation of cells from chorionic plate has been recently documented (Kawamichi et al., 2010) using a standard chemical treatment of inducing myoblasts formation (5-azacytidine) and also forcing *MYOD* expression through a lentiviral vector (Akizawa et al., 2013). Kawamichi and colleagues proved also the *in vivo* ability of placenta-derived cells to ameliorate the dystrophic phenotype of *mdx* mice inducing the appearance of newly formed fibers expressing human dystrophin.

### Amniotic fluid stem cells

Amniotic fluid contains a heterogeneous population of cells displaying a wide range of morphologies. Most of these cells are epithelial in nature and have a limited capacity to proliferate in culture. Traditionally, AF has been used for decades as a tool for prenatal diagnosis, but recent studies provided important evidences about the potential of AF as an alternative source of stem cells. Many works have characterized putative stem cell populations isolated from AF, such as Prusa et al. (2003) that showed the expression of OCT4 within a subset of AF cells. Moreover, demonstration of proliferation within this population suggests that pluripotent stem cells can be isolated and propagated from the human AF.

Employing immunoselection technique, AF cells expressing the cell surface antigen c-Kit were purified from primary amniocentesis cultures (De Coppi et al., 2007). Isolated cells, called AFS cells, grew rapidly in culture, display a normal karyotype and maintain telomere length during long-term culture. This latter attribute facilitated the establishment of clonal lines from AFS cells, necessary to establish the “stemness” of a population. Clonal AFS cell lines differentiated *in vitro* to putative adipocytes, endothelial cells, hepatocytes, osteocytes, myocytes, and neurons, derivatives of all germ layers. This broad plasticity appeared to be a general attribute of the selected cells: 19 different amniocentesis cultures yielded multipotent AFS cell clonal lines. A subsequent report demonstrated the AFS cells *in vitro* chondrogenic differentiation thus providing further evidence of the plasticity and clinical potential of cells isolated from the AF (Preitschopf et al., 2012). Moreover, these cells displayed the ability to reconstitute the depleted bone marrow of *Rag1*^−/−^ mice after systemic injection and secondary transplantation (Ditadi et al., 2009).

Another peculiar characteristic of AFS cells is indeed the ability to pass through the endothelial barrier, thus they can be administered locally or systemically, which represents a great advantage in treating whole body diseases such as muscle dystrophies. Considering this important aspect, mouse AFS cells were used in the *HSA-Cre*, *Smn^F7/F7^* mouse model by transplantation via tail vein (Piccoli et al., 2012). After injection, AFS cells demonstrated remarkable ability to differentiate directly *in vivo* into myogenic cells but most importantly were able to generate new muscle fibers after cardiotoxin injury or secondary transplant, suggesting the idea that AFS cells are capable of differentiating also in skeletal muscle stem cells repopulating the muscle niche. In this work, the use of freshly isolated or expanded murine AFS cells generated similar results, but it is worth noticing that, with human AFS cells, muscle lineage differentiation becomes difficult to obtain after *in vitro* expansion. Gekas and colleagues indeed, did not find any myogenic differentiation of human AF c-Kit^+^ cells after transplantation into the skeletal muscle of SCID mice (Gekas et al., 2010). Myogenic differentiation of AF cells can be induced *in vitro* by DNA demethylation (e.g., using chemicals such as 5-aza-2′-deoxycytidine), co-culture with myoblasts or myogenic cell lines, or directly with *in vivo* muscle transplantation (De Coppi et al., 2007; Gekas et al., 2010; Ma et al., 2012; Yang et al., 2012), but all these approaches result in the induction of MYOD expression. In fact, by transfecting human AFS cells with *MYOD* lentivirus, Kim and co-workers demonstrated that human cells also were able to differentiate into myogenic lineage *in vitro* and *in vivo*, after injection into injured muscles of immunodeficient BALB/cSlc-nu mice (Kim et al., 2013). All these data indicate that stem cells obtained from discarded AF could be considered good candidates for deeper research and investigation on their ability to differentiate into myogenic cells. Further analyses must be performed to make these cells more committed and at the same time safe for future clinical applications using, for instance, non-viral system such as Piggy Bac or Sleeping Beauty transposons (Izsvak et al., 2009). These tools allow modifying cells by inserting new genes, ensuring a constitutive genetic integration of the insertion and opening possible way to cure genetic defects.

## Conclusion

Muscle pathologies are devastating diseases and nowadays researchers still make efforts to find a cure and not a therapy alone. It has been demonstrated that, after injection in injured or diseased muscle, fetal stem cells act through a mechanism that is mostly due to a bystander effect rather than a direct differentiation. The indirect action is mainly supposed to enhance the production of cytokines, such as VEGF, that stimulate the temporary restoring of the tissue function. To obtain a long lasting action due to efficient cell integration and tissue repopulation, fetal stem cells need to be genetically modified, forcing their differentiation in tissue-specific cells. Nevertheless, the development of safe genetic manipulation methods could make cells of fetal origin appealing for therapeutic application.

Conversely, the long-term positive effect observed using freshly isolated murine AFS cells, highlights that they could have a decisive role in replenishing the muscle stem cell niche, which represent the reservoir of cells able to rescue the defect. Indeed, AFS cells are a safe and immune-privileged cell source prone to integrate in muscle tissue. This knowledge opens the challenge to improve the culture protocol for the AFS cells of human origin, which, so far, is still a limit to overcome for future clinical application to treat genetic and non-genetic muscle dysfunctions (dystrophies, skeletal muscle malformations, traumatic injuries).

## Conflict of Interest Statement

The authors declare that the research was conducted in the absence of any commercial or financial relationships that could be construed as a potential conflict of interest.
